# Supplementary figures and images for: Neurodevelopmental Outcome of Very Low Birth Weight Infants in the Northern District of Israel: A Cross-Sectional Study
Source: Children (Basel). 2023 Jul 31;10(8):1320. doi: 10.3390/children10081320 (PMC10453082; doi:10.3390/children10081320)

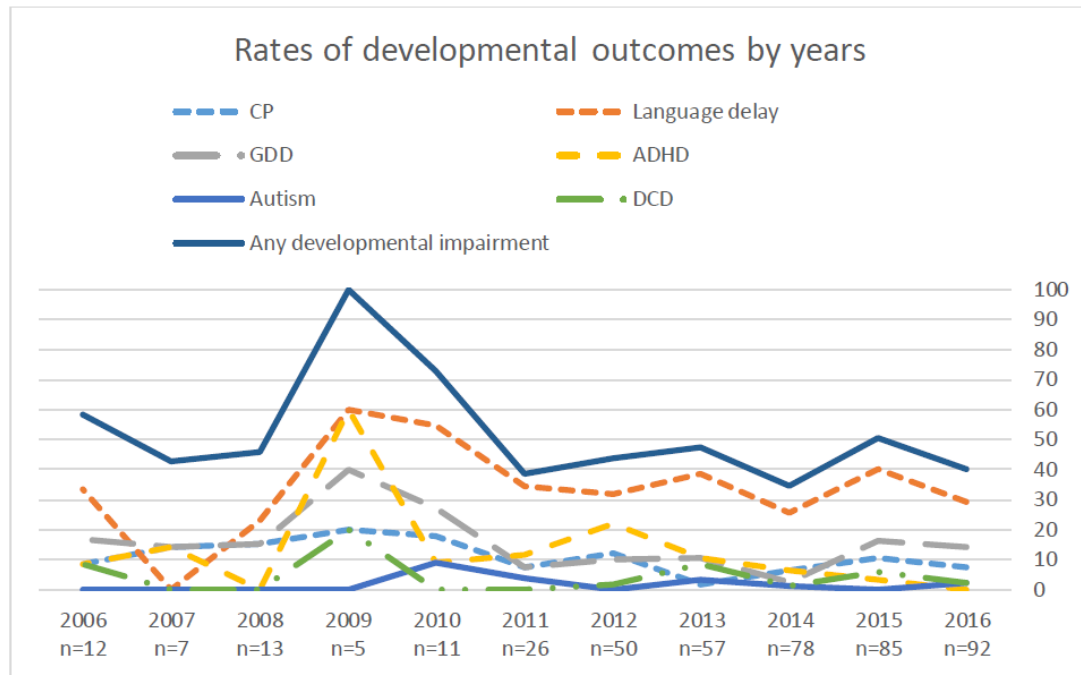

**Figure S1.** Rates of developmental outcomes by years.

Supplement: Supplementary file 1 [file children-10-01320-s001.zip › children-2490744-supplementary.pdf]
